# Effective adaptation to rising flood risk

**DOI:** 10.1038/s41467-018-04396-1

**Published:** 2018-05-29

**Authors:** Brenden Jongman

**Affiliations:** 1 Institute for Environmental Studies, VU University Amsterdam, Amsterdam, The Netherlands; 2 Global Facility for Disaster Reduction and Recovery (GFDRR), The World Bank, Washington, DC USA

Floods are causing increasing havoc in our rapidly urbanizing world, with disproportionally high impacts on the poorest and most vulnerable. Effective adaptation strategies are needed, which combine flood protection infrastructure, nature-based solutions, and risk financing schemes to manage floods and buffer their economic impacts.

Global weather-related disaster losses exceeded US$300 billion in 2017, which made this the most costly year on record and continues a long-term upward trend^1^. For the first time in history, over half the world’s population now live in cities, many of which are located at rivers, along coastlines, or both. A new study shows that the total urban area exposed to flooding in Europe has increased by 1000% over the past 150 years^2^. On a global scale, trends in flood zone urbanization have been similarly steep and continue to climb, especially in Africa and Asia^3,4^. Not only does this mean that ever more human assets are in the way of floods, but urbanization with an increase of non-permeable surfaces and lack of natural drainage creates additional flooding issues that did not previously exist.

Socioeconomic changes are further compounded by climate change-induced increases in extreme rainfall which amplify the intensity and probability of floods. In Europe, all climate models consistently show an increase in flood impacts across most Western and Central countries, approximately doubling the expected damage by the end of the century^5^. In addition, changing patterns of spring snowmelt and winter storms have dramatically affected the timing of floods throughout the year^6^. Within these long-term trends, inter-annual fluctuations due to large scale atmospheric patterns such as the North Atlantic Oscillation (NAO) and the East Atlantic pattern (EA) cause strong year-to-year fluctuations in rainfall and resulting flood damages^7^.

The impacts of flooding go far beyond direct damages to assets and infrastructure. Economic losses resulting from business disruption, welfare effects and supply chain shocks can often at times equal or exceed direct damages^8^. In extreme cases, such as the shut-down of Wall Street due to Hurricane Sandy, economic ripple effects may be felt across an entire sector around the globe. The welfare loss from flood events hits the poorest in society hardest. In many countries, the poorest population groups are relatively overexposed to flooding, as they are often forced to live and work in low-lying areas^9^. In addition, the poorest households are more vulnerable to the resulting impacts to their income, and can often be pushed across the poverty line by a single event^10^. As such, natural disasters may effectively increase global poverty^11^.

Positive trends, however, are visible. Economic development, technological progress and targeted adaptation interventions help reduce flood impacts over time. In Europe, fatalities and normalized economic losses (losses as % of GDP) have decreased significantly over recent decades, despite an increase in flooded area and absolute losses^2^. Globally, too, such a decline in relative impact has been observed, as low-income countries are becoming less vulnerable as per-capita income rises^12^. This demonstrates that effective adaptation to flooding is feasible, even when faced by growing exposure and a changing climate.

## Adaptation to flooding

If all natural disasters could suddenly and completely be eliminated, hundreds of billions of dollars in damages would be saved each year and the number of people living in extreme poverty would immediately fall by 26 million^11^. However, not all disasters can be prevented. Effective adaptation to rising flood risk requires a diversified approach of interventions, which may include structural flood protection measures, early warning systems, risk-informed land planning, nature-based solutions, social protection and risk financing instruments^13^. The right mix of measures varies from place to place, subject to levels of risk, funding, and political will.

Physical flood protection measures, such as dikes and levees, are generally cost-effective in areas with high population and asset concentrations^14^. The Netherlands, being a highly populated and highly flood prone country, is the prime example of a country relying heavily on such structural measures. Most of the coastline is protected with a dike system offering protection against events that only occur once every 10,000 years. However, such protection works require immense capital investments for construction and maintenance, for which both political momentum and government budgets are often missing. In addition, research in the field of socio-hydrology has shown that increasing flood protection can give a false sense of security and may boost development in these protected flood-prone areas. However, while the resulting system may have a lower risk overall, the potential impacts of a dike-breaching event can be catastrophic^15^.

Recently, governments are increasingly turning to nature to manage flooding^16^. Such nature-based solutions include widening of natural flood plains, protecting and expanding wetlands, restoring oyster and coral reefs and investing in urban green spaces to reduce run-off. In the United States, natural wetlands have moderated damages from Hurricane Sandy by an estimated $625 million^17^. In the Gulf of Mexico, nature-based adaptation measures could even reduce overall risk by a stunning $50 billion, with an average benefit to cost ratio of 3.5^18^. Meanwhile, China has started implementing the national “Sponge Cities” program in 16 pilot cities, where vast amounts of green space will be integrated into urban design to prevent surface flooding. In addition to effectively reducing flood risk, nature-based solutions can have a wide range of positive effects on ecosystem conservation, carbon storage, tourism and local employment. Implementing natural approaches often also requires the involvement of various stakeholder groups, thereby helping with awareness raising and consensus building.

**Fig. 1:**
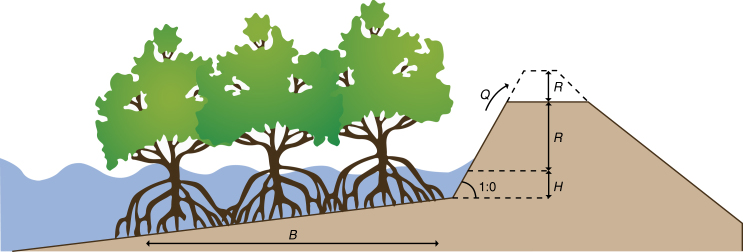
Hybrid flooding solutions. By reducing wave run-up, a hybrid solution that combines mangrove conservation with a levee can reduce construction and maintenance costs while offering the same protection level. Figure reproduced from ref. ^20^

Yet, in spite of the multiple benefits of nature-based solutions, flood control remains heavily dependent on ‘gray’ infrastructure interventions. The relatively slow uptake of nature-based solutions demonstrates the current lack of understanding regarding feasible protection levels, appropriate maintenance and monitoring schemes, and available flexible funding mechanisms for such approaches^19^. In many cases, combining green and gray infrastructure measures into so-called ‘hybrid’ solutions have the best potential to safeguard the security provided by infrastructure while providing the benefits of natural approaches (Fig. 1). Careful analyses should be conducted to evaluate the range of available options and design flood management schemes that combine natural, infrastructural and policy instruments in the most effective way^20^.

## Financing the residual risk

Whereas adaptation has ensured that normalized losses as a percentage of GDP are stable, or even declining in some regions, the absolute financial loss levels are still on the rise. Financing the repair of and recovery from flood damages has therefore become a rising challenge. Uninsured losses and the lack of financial means for flood recovery and response may impact peoples’ well-being, the country’s budget and the overall economy. The construction of risk financing strategies determines the short-term and long-term financial burden of floods on individuals and companies. If effectively designed, risk transfer mechanisms such as insurance products can even incentivize active risk reduction by policy holders.

Individual European countries have put in place a variety of national risk financing systems, including differing private flood insurance products. However, the insurance coverage rates are generally low, and cannot work independently without a parallel government mechanism. Government support can include subsidizing insurance premiums, managing insurance schemes, acting as a monopolistic insurance provider, or simply providing ad-hoc post-disaster aid^21^. The latter is often associated with negative effects, such as reduced incentives for risk reduction.

On a continental level, the European Commission also provides financial relief through the EU Solidarity Fund. This Fund can supplement the financial expenditures of member states for large events. However, the Solidarity Fund is insufficient to cover all eligible losses and does not encourage risk reduction^22^. Under climate change induced increases in flood extremes, the financial sustainability of the Fund is likely to deteriorate further. Europe, however, is not the only place where financing schemes are dependent on accurate understanding of risk trends. In the United States, new modeling work has shown that the official national flood maps may underestimate flood exposure by a factor 3, which could have important consequences for the national insurance program^23^.

## Risk perception

The design of such holistic risk management strategies requires an accurate understanding of the level of risk across the various layers of society. One important remaining limitation in our understanding of flood risk is the way individuals perceive and respond to risk. Even if we manage to model population density and flood inundation with increasing accuracy, assumptions about peoples’ risk reducing behavior, willingness to relocate, and access to information play a key role in the actual level of risk. Neglecting this behavioral component may overestimate actual impacts by a factor two^25^. Recent innovations in agent-based modeling now allow us to integrate complex human behavior in integrated risk assessments, which will allow us to create much more realistic scenarios of flood impacts and possible adaptation solutions^26^.

**Fig. 2:**
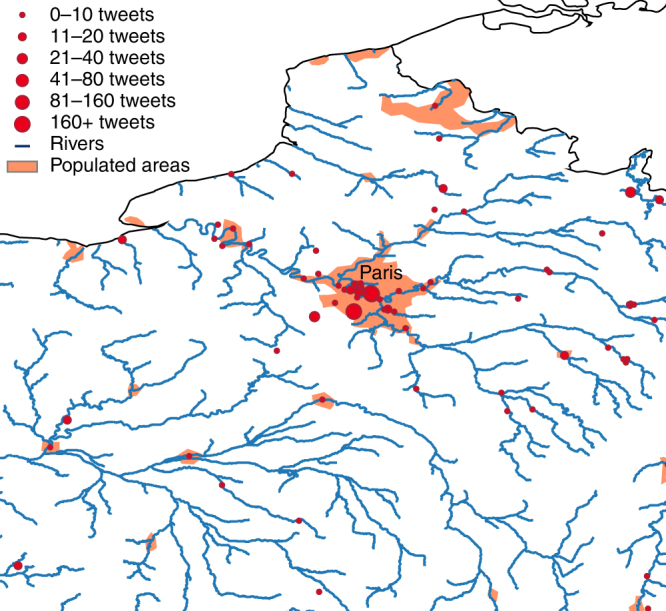
Social media and flooding. Number of flood-related Twitter messages (“tweets”) during the January 2018 floods in France. Advances in social media analytics are used to detect and monitor floods earlier, allowing for more effective response (figure produced by the author using data from the TAGGS^24^ model, accessible through the Global Flood Monitor - https://www.globalfloodmonitor.org/)

The new age of social media may help us better understand the human aspects of flooding. All Twitter messages are now automatically screened and georeferenced in order to identify and locate flood events around the world^24^ (Fig. 2). The Philippines Red Cross is already using this as an operational tool for flood response, opening the door not only to the earlier detection of events, but also to understanding the perception, impacts and response to floods as they occur. In areas where Twitter is not available, similar rapid text-mining technologies are currently being developed that rely on websites, newspaper articles, and text messages.

Clearly, managing flood risk involves much more than building dikes. Governments need to be invested in the complex task of adopting holistic risk management strategies that combine economically viable investments in risk reduction along with well-designed financial instruments to cover residual losses, whilst acknowledging the ever-changing and cross-boundary nature of risk.

## References

[1] Swiss Re. *Preliminary sigma catastrophe estimates for 2017*. (2017).

[2] Paprotny, D., Sebastian, A., Morales-Napoles, O. & Jonkman, S. Trends in flood losses in Europe over the past 150 years. *Nat. Commun*. (2018). 10.1038/s41467-018-04253-1 PMC5974183 29844471

[3] Winsemius HC (2015). Global drivers of future river flood risk. Nat. Clim. Chang.

[4] Jongman B, Ward PJ, Aerts JCJH (2012). Global exposure to river and coastal flooding: long term trends and changes. Glob. Environ. Chang.

[5] Alfieri L, Dottori F, Betts R, Salamon P, Feyen L (2018). Multi-model projections of river flood risk in Europe under global warming. Climate.

[6] Blöschl G (2017). Changing climate shifts timing of European floods. Science.

[7] Guimares Nobre, G., Jongman, B., Aerts, J. & Ward, P. J. The role of climate variability in extreme floods in Europe. *Environ. Res. Lett*. **12**, 084012 (2017).

[8] Hallegatte S (2008). An adaptive regional input-output model and its application to the assessment of the economic cost of katrina. Risk Anal..

[9] Winsemius, H. C. et al. Disaster risk, climate change, and poverty: assessing the global exposure of poor people to floods and droughts. *Environ. Dev. Econ*. **7**, 642–646 (2018).

[10] Hallegatte, S. et al. *Shock waves: managing the impacts of climate change on poverty*. (The World Bank, 2015).

[11] Hallegatte, S., Vogt-Schilb, A., Bangalore, M. & Rozenberg, J. *Unbreakable: building the resilience of the poor in the face of natural disasters.* (World Bank Publications, 2017).

[12] Jongman B (2015). Declining vulnerability to river floods and the global benefits of adaptation. PNAS 1414439112-.

[13] Aerts JCJH, Botzen WJW, Emanuel K, Lin N, Moel HDe (2014). Evaluating Flood Resilience Strategies for Coastal Megacities. Sci. (80-.).

[14] Ward, P. J. et al. A global framework for future costs and benefits of river-flood protection in urban areas. *Nat. Clim. Chang*. **17**, 642-646 (2017).

[15] Ciullo A, Viglione A, Castellarin A, Crisci M, Di Baldassarre G (2017). Socio-hydrological modelling of flood-risk dynamics: comparing the resilience of green and technological systems. Hydrol. Sci. J..

[16] Temmerman S (2013). Ecosystem-based coastal defence in the face of global change. Nature.

[17] Narayan S (2017). The Value of Coastal Wetlands for Flood Damage Reduction in the Northeastern USA. Sci. Rep..

[18] Reguero BG, Beck MW, Bresch DN, Calil J, Meliane I (2018). Comparing the cost effectiveness of nature-based and coastal adaptation: A case study from the Gulf Coast of the United States. PLoS One.

[19] UN-Water. *The United Nations World Water Development Report 2018: Nature-Based Solutions for Water*. (2018).

[20] van Wesenbeeck, B. K. et al. *Implementing Nature Based Flood Protection: Principles and Implementation Guidance*. World Bank Group, Washington, D.C., (2017).

[21] Prettenthaler F, Albrecher H, Asadi P, Köberl J (2017). On flood risk pooling in Europe. Nat. Hazards.

[22] Jongman B (2014). Increasing stress on disaster-risk finance due to large floods. Nat. Clim. Chang.

[23] Wing OEJ (2018). Estimates of present and future flood risk in the conterminous United States. Environ. Res. Lett..

[24] de Bruijn JA, de Moel H, Jongman B, Wagemaker J, Aerts JCJH (2018). TAGGS: Grouping Tweets to Improve Global Geoparsing for Disaster Response. J. Geovisualization Spat. Anal..

[25] Haer T, Botzen WJW, de Moel H, Aerts JCJH (2017). Integrating Household Risk Mitigation Behavior in Flood Risk Analysis: An Agent-Based Model Approach. Risk Anal..

[26] Aerts JCJH (2018). Integrating human behaviour dynamics into flood disaster risk assessment. Nat. Clim. Chang.

